# High Prevalence of Illicit Drug Use in Men Who Have Sex with Men with HIV-1 Infection in Japan

**DOI:** 10.1371/journal.pone.0081960

**Published:** 2013-12-10

**Authors:** Takeshi Nishijima, Hiroyuki Gatanaga, Hirokazu Komatsu, Misao Takano, Miwa Ogane, Kazuko Ikeda, Shinichi Oka

**Affiliations:** 1 AIDS Clinical Center, National Center for Global Health and Medicine, Tokyo, Japan; 2 Center for AIDS Research, Kumamoto University, Kumamoto, Japan; 3 Department of Community Care, Saku Central Hospital, Nagano, Japan; Temple University School of Medicine, United States of America

## Abstract

**Objective:**

To examine the prevalence of illicit drug use among men who have sex with men (MSM) with HIV-1 infection in Japan, where the life-time prevalence of illicit drug use in the general population is only 2.9%.

**Design:**

A single-center cross-sectional study at a large HIV clinic in Tokyo, which treats approximately 15% of HIV-1 infected patients in Japan.

**Methods:**

The prevalence of illicit drug use and the assciation of characteristics and social demographics of the patients with illicit drug use were examined. Patients who visited the clinic for the first time from 2005 to 2010 were enrolled. Relevant variables were collected using a structured interview and from the medical records. Multivariate logistic regression analyses were applied to estimate the odds of association of MSM over non-MSM HIV-infected patients with illicit drug use.

**Results:**

1,196 patients were enrolled. They were mostly Japanese men of relatively young age. Illicit drug use (including injection drugs) was reported by 35% of the patients (by 40% of MSM), and 4% were IDU while 5% were on methamphetamine. 2% of the population was arrested due to illicit drugs. MSM was significantly associated with illicit drug use (adjusted OR = 4.60; 95% CI, 2.88–7.36; p<0.01). Subgroup analysis of the patients stratified by three age groups (≤30, 31 to 40, and >40) showed that the odds of association of MSM with illicit drug use was the strongest in the youngest age group (≤30 years: adjusted OR = 7.56; 95% CI, 2.86–20.0; p<0.01), followed by the oldest (>40 years: adjusted OR = 6.15; 95% CI, 2.40–15.8; p<0.01), and the weakest in the group aged 31 to 40 (adjusted OR = 3.39; 95% CI, 1.73–6.63; p<0.01).

**Conclusions:**

The prevalence of illicit drug use is high among MSM patients with HIV-1 infection in Japan. Effective intervention for illicit drug use in this population is warranted.

## Introduction

Illicit drug users, especially injection drug users (IDU), are at high risk of infection with HIV-1 [1], [2]. They are one of the “difficult to reach” populations, especially with regard obtaining accurate prevalence data [3]. In Japan, the prevalence of illicit drug use in the general population is only 2.9% according to the 2009 Nationwide General Population Survey on Drug Use and Abuse [4], [5] (http://www.ncnp.go.jp/nimh/pdf/h21.pdf. in Japanese) (http://www.mhlw.go.jp/bunya/iyakuhin/yakubuturanyou/torikumi/dl/index-04.pdf. in Japanese). To our knowledge, however, no study has examined the prevalence of illicit drug use among patients with HIV-1 infection in Japan.

Among patients with HIV-1 infection, illicit drug use is associated with lower antiretroviral therapy (ART) uptake and inferior adherence [6]–[9], which leads to suboptimal treatment outcome, compared with patients with other risk categories [10]–[12]. The aim of the present study was to examine the prevalence of illicit drug use in patients with HIV-1 infection and its association with characteristics of the patients in Japan, in order to establish effective intervention strategies.

## Methods

### Ethics Statement

This study was approved by the Human Research Ethics Committee of National Center for Global Health and Medicine, Tokyo, Japan. The Committee waived a written informed consent, because this study only used data of patients from routine clinical practice. However, at our clinic each patient provided a written informed consent for the clinical and laboratory data to be used and published for research purposes [13]. We conducted this study according to the principles expressed in the Declaration of Helsinki.

### Study design

This study was designed and reported according to the recommendations of Strengthening the Reporting of Observational studies in Epidemiology (STROBE) statement [14]. We performed a single center cross-sectional study of patients with HIV-1 infection to examine the prevalence of illicit drug according to patient characteristics including sexual orientation, primarily focusing on men who have sex with men (MSM). Illicit drugs were defined as legally prohibited substances in Japan; They included amyl nitrite and 5-methoxy-diisopropyltryptamine, which became prohibited by law in 2006 and 2005, respectively, in Japan [15]. This study was conducted at the AIDS Clinical Center, Tokyo. Our facility is one of the largest clinics for HIV care in Japan with more than 3,300 registered patients [13]. Considering that the total reported number of patients with HIV-1 infection is 21,415 by the end of 2011, this clinic treats approximately 15% of the HIV-1 infected patients in Japan (http://api-net.jfap.or.jp/status/2011/11nenpo/hyo_02.pdf. in Japanese).

### Study Subjects

The study population comprised patients with HIV-1 infection, aged >17 years, who visited our clinic for the first time from January 1, 2005 to August 31, 2010. The following exclusion criteria were applied; 1) those who visited the clinic for a second opinion, 2) those referred to other facilities on their first or second visit. These patients were excluded because the structured interview on social demographics was often not conducted in these patients, 3) patients infected through contaminated blood products (e.g. hemophiliacs) and mother to child transmission, and 4) patients who refused to be included in the study.

### Measurements

Variables were collected through a structured interview conducted at the first visit as part of routine clinical practice by the nurses specializing at the HIV outpatient care. The interview by these “coordinator nurses” included the following variables: history of illicit drug use and injection drug use (and their types if available), perceived route of transmission, sexual orientation (men were asked whether they have sex with men), history of gay bathhouse use (if MSM), working status, and living status (alone or with someone else) [16]. Because interviews could potentially underestimate the prevalence of illicit drug use, we also searched the medical records for information on illicit drug use and related variables covering the period from the first visit to December 2012. Data of age, sex, ethnicity, current treatment status for HIV infection, and history of AIDS (defined as history of or concurrent 23 AIDS-defining diseases set by the Japanese Ministry of Health, Labour and Welfare) were obtained from the medical records (http://www.haart-support.jp/pdf/guideline2012.pdf in Japanese). The laboratory data of CD4 cell count, HIV-1 viral load, hepatitis C antibody on the first visit were also collected, and when these tests were not conducted on that day, data within three months from the first visit were used.

### Statistical analysis

Patients' characteristics and social demographics were compared between MSM and non-MSM groups by the Student's t-test for continuous variables and by either the χ^2^ test or Fisher's exact test for categorical variables. Logistic regression analysis was used to estimate the odds of association of MSM, relative to non-MSM, with illicit drug use. The odds of association of each basic demographics, baseline laboratory data, and other medical conditions listed above was also estimated with univariate analysis.

To estimate the odds of association of MSM over non-MSM with illicit drug use, we conducted multivariate logistic regression analysis adjusted by age and ethnicity. Age and ethnicity (Japanese) were selected among four variables with *p* value <0.05 in univariate analysis, because age is a basic demographic and the literature had reported that population/ethnicity can affect the prevalence of illicit drug use [17]. The two variables; “ART” and “history of AIDS” were not included because they were not considered to be related to illicit drug use.

To estimate the odds of association of different age categories with illicit drug use, we divided the group into three age subgroups: ≤30, 31 to 40, and >40 years. Then, the abovementioned multivariate analysis was conducted for each subgroup.

Statistical significance was defined at two-sided *p* value of <0.05. We used odds ratios (ORs) and 95% confidence intervals (95% CIs) to estimate the odds of association of each variable with illicit drug use. All statistical analyses were performed with The Statistical Package for Social Sciences ver. 20.0 (SPSS, Chicago, IL).

## Results

**Figure 1:**
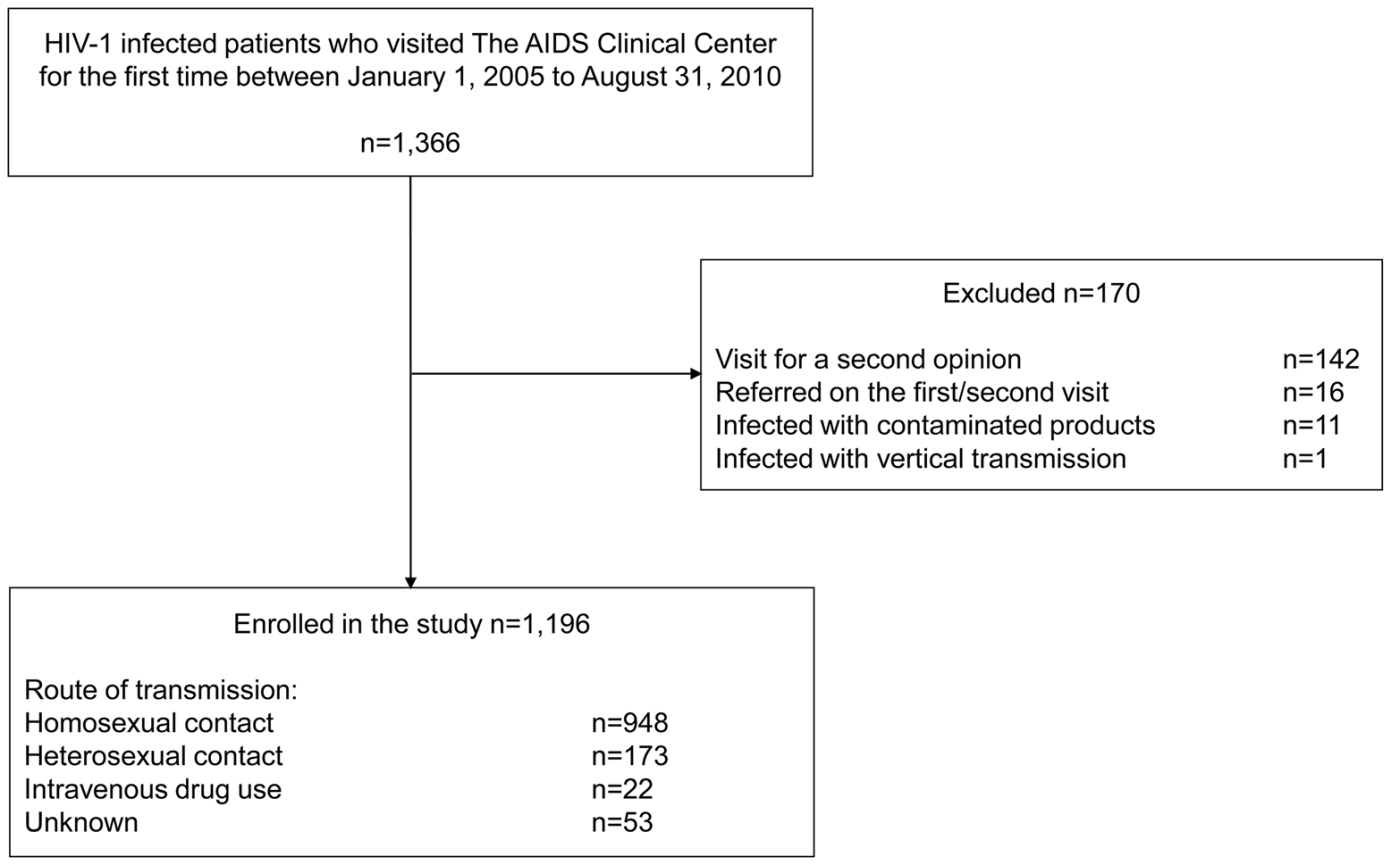
Patient enrollment.

During the study period, 1,366 patients with HIV-1 infection visited the AIDS Clinical Center for the first time, and 170 patients were excluded from the analysis based on the abovementioned exclusion criteria (Figure 1). For the 1,196 patients included in the study, the perceived route of transmission was male-to-male sexual contact in 948 (79%), heterosexual contact in 173 (14%), IDU in 22 (2%), and unknown in 53 (4%). The majority of the study patients were relatively young Japanese men with a median age of 36 years. Most patients were ART-naïve, with a median CD4 count of 245/µl (Table 1).

**Table 1:** Baseline characteristics of total study patients, MSM, and non-MSM.

	Total (n = 1,196)	MSM (n = 973)	Non-MSM (n = 223)	P value
Sex (male), n (%)	1,114 (93)	973 (100)	152 (63)	<0.01
Age (years)†	36 (29–43)	35 (29–42)	38 (31–47)	<0.01
History of illicit drug use, n (%)	415 (35)	392 (40)	23 (10)	<0.01
Injection drug use, n (%)	53 (4)	44 (5)	9 (4)	0.73
Methamphetamine use, n (%)	63 (5)	57 (6)	6 (3)	0.07
Arrested due to illicit drugs, n (%)	27 (2)	26 (3)	1 (0.4)	0.04
History of gay bathhouse use, n (%)	Not applicable	461 (47)		
Ethnicity, n (%)^a^				
Japanese	1058 (89)	906 (93)	152 (68)	<0.01
Asian	70 (6)	29 (3)	41 (18)	
White	27 (2)	26 (3)	1 (0.4)	
Black	26 (2)	2 (0.2)	24 (11)	
Latino	12 (1)	7 (1)	5 (2)	
Working status, n (%)^b^				
Without job	226 (19)	163 (17)	63 (28)	<0.01
Working	902 (75)	763 (78)	139 (63)	
Student	56 (5)	47 (5)	9 (4)	
Housewife	11 (1)	0	11 (5)	
Living alone, n (%)^c^	530 (44)	475 (49)	55 (25)	<0.01
CD4 count (/µl)† ^d^	245 (101–379)	252 (114–380)	207 (50–379)	0.08
HIV-1 viral load (log_10_/ml)† ^e^	4.60 (3.91–5.20)	4.64 (3.94–5.20)	4.43 (3.26–5.08)	<0.01
On antiretroviral therapy, n (%)	120 (10)	85 (9)	35 (16)	<0.01
History of AIDS, n (%)	321 (27)	247 (25)	74 (33)	0.02
Positive HCV antibody, n (%)	38 (3)	19 (2)	19 (9)	<0.01

median (interquartile range).

^a^three, ^b^one, ^c^fifteen, ^d^two, and ^e^four patients were not available (missing). Data of

Among the 1,196 patients, 415 (35%) had used or were illicit drug users, and 53 (4%) were IDUs while 63 (5%) reported using methamphetamine. With regard to social history, 27 (2%) had been detained or arrested due to possession or use of illicit drugs (Table 1). Among the illicit drugs used, amyl nitrite and 5-methoxy-diisopropyltryptamine were the most commonly named by the patients. 3,4-methylenedioxymethamphetamine, cannabis, heroin, cocaine, and opium were also mentioned (numbers not counted except for methamphetamine).

Of the 1,196 patients, 973 (81%) were MSM regardless of the perceived route of transmission (e.g., if a patient considered to have been infected with HIV-1 through injection drug use and was MSM, he was classified as MSM in Table 1). Compared with non-MSM patients, MSM were significantly younger and more likely to be Japanese. MSM patients were more likely to have experienced illicit drugs [392 (40%)] than non-MSM [23 (10%), p<0.01], and have used methamphetamine [57 (6%) versus 6 (3%), p = 0.07], and to have been arrested/detained due to illicit drug use/possession [(26 (3%) versus 1 (0.4%), p = 0.04) (Table 1). There was no difference in the percentage of IDUs among the MSM and non-MSM groups [44 (5%) versus 9 (4%), p = 0.73]. The CD4 count of MSM patients tended to be higher, and MSM were less likely to present with AIDS than non-MSM, although HIV viral load of MSM was significantly higher than that of non-MSM. MSM were more likely to have a job and be living alone. Further analysis showed that 47% of MSM patients used a gay bathhouse, and among them, the prevalence of illicit drug use was higher (49%) than all MSM (40%). The prevalence of illicit drug use was even higher in MSM aged ≤30 years (52%).

Univariate analysis showed a significant relationship between MSM and illicit drug use (OR = 5.87; 95% CI, 3.74–9.20; p<0.01) (Table 2, Model 1). Furthermore, younger age, being Japanese, on ART, and history of AIDS were associated with illicit drug use. On the other hand, without a job, living alone, and positive HCV antibody were not associated with illicit drug use. Multivariate analysis identified MSM to be significantly associated with illicit drug use after adjustment for age and Japanese (adjusted OR = 4.60; 95% CI, 2.88–7.36; p<0.01) (Table 2, Model 2).

**Table 2:** Results of multivariate analysis of the association of MSM over non-MSM for illicit drug use.

	Model 1 Crude n = 1,196		Model 2 Adjusted n = 1,196	
	OR	95% CI	OR	95% CI
Men who have sex with men†	5.87	3.74–9.20	4.60	2.88–7.36
Age per 1 year†			0.95	0.94–0.97
Japanese†			1.74	1.07–2.82

p<0.05.

Subgroup analysis of the patients stratified by three age groups (≤30, 31 to 40, and >40) showed that the odds of association of MSM with illicit drug use was the strongest in the youngest age group (≤30 years: adjusted OR = 7.56; 95% CI, 2.86–20.0; p<0.01), followed by the oldest (>40 years: adjusted OR = 6.15; 95% CI, 2.40–15.8; p<0.01), and the weakest in the group aged 31 to 40 (adjusted OR = 3.39; 95% CI, 1.73–6.63; p<0.01) (Table 3).

**Table 3:** Results of multivariate analysis of the association of MSM over non-MSM for illicit drug use according to age.

	Adjusted OR	95% CI	P value
Age ≤30 years (n = 369)			
MSM vs. non-MSM	7.56	2.86–20.0	<0.01
Age 31 to 40 years (n = 473)			
MSM vs. non-MSM	3.39	1.73–6.63 85	<0.01
Age >40 years (n = 354)			
MSM vs. non-MSM	6.15	2.40–15.8	<0.01

Table 2. MSM was adjusted with the same variables as Model 2,

MSM: men who have sex with men.

## Discussion

The prevalence of illicit drug use among patients with HIV-1 infection in this large urban HIV clinic in Tokyo, which treats approximately 15% of patients with HIV-1 infection in Japan, was high at 35%. The prevalence was higher among HIV-1 infected MSM (40%), especially among young MSM aged ≤30 years (52%). Furthermore, HIV-1 infected MSM were more likely to use methamphetamine and to be arrested due to illicit drugs, compared with non-MSM. It should be emphasized that these numbers are likely to be underreported, since some patients would not admit illicit drug use to the interviewers on their first visit.

To our knowledge, this is the first study on the prevalence of illicit drug use among patients with HIV-1 infection in Japan. Although the prevalence of illicit drug use is considered extremely low among the general population in Japan with lifetime prevalence of 2.9% in 2009, high prevalence of illicit drug use in patients with HIV-1 infection, especially among HIV-1 infected MSM, was demonstrated [4], [5] (http://www.ncnp.go.jp/nimh/pdf/h21.pdf. in Japanese) (http://www.mhlw.go.jp/bunya/iyakuhin/yakubuturanyou/torikumi/dl/index-04.pdf. in Japanese). The prevalence of methamphetamine use and incarceration due to illicit drug was also high, suggesting a substantial impact of illicit drugs, not only on the well-being of this population in terms of both medical and social perspectives, but also on public health perspectives [11], [12].

In Japan, the number of illicit drug users arrested in 2010 was 14,965. Among these, 12,200 used methamphetamine, followed by cannabis (2,367), while only several hundred at most used other drugs (http://www.mhlw.go.jp/bunya/iyakuhin/yakubuturanyou/torikumi/dl/index-01.pdf in Japanese). Of note, the number of arrestees due to other injectable drugs, such as heroin and cocaine, was small (22 and 112, respectively). Thus, most injection drug users in Japan are methamphetamine users. Majority of the patients identified as IDU in this study were considered to be methamphetamine users as well.

By the end of 2011, of 19,976 patients (excluding those infected with contaminated blood products) reported to be infected with HIV-1, 108 (0.5%) were reported to be infected through injection drug use according to the surveillance conducted by the AIDS Surveillance Committee of the Japanese Ministry of Health, Labour and Welfare (http://api-net.jfap.or.jp/status/2011/11nenpo/hyo_02.pdf in Japanese). The prevalence of IDUs in this study is substantially higher; 53 (4%) of the 1,196 were IDUs, suggesting a considerable underreporting of IDU in the surveillance data. It is well known that for IDUs, prognosis is much worse than non-injecting drug users, as one multicenter study conducted in Europe and North America reported that IDUs experienced approximately five times higher mortality rates than patients infected through sexual intercourse [18]. Although the prevalence of IDUs among patients with HIV-1 infection in Japan is still much lower than that in neighboring countries, such as Taiwan (27.6%) and China (24.3%), there is an urgent need to develop effective prevention programs for HIV-1 infected illicit drug users [19] (http://www.unaids.org.cn/download/2009%20China%20Estimation%20Report-En.pdf) (http://www.cdc.gov.tw/english/list.aspx?treeid=00ED75D6C887BB27&nowtreeid=334C2073091C8677).

Although the prognosis of injection drug users is reported to be worse than that of non-injection drug users [20], this study primarily focused on illicit drug use as a whole, rather than injection drug use. This is because only a few studies focused on illicit drug use among HIV-1 infected patients, although a large number of studies focused on injection drugs [21]–[25]. Illicit drug use in patients with HIV-1 infection is an important issue, because not only illicit drug use lead to inferior treatment outcome compared with non users [10]–[12], but also non injection drug users are prone to practice high risk sexual behaviors, which might lead to transmission of HIV and other infectious diseases [8], [26]. Studies from the US reported that especially MSM who use illicit drugs are at high risk for HIV and sexual transmitted infections due to close associations between risky sexual behaviors and illicit drug use [27], [28] Furthermore, illicit drug use, especially opioid use, can be a trajectory into injection drug use [29], [30].

Several limitations need to be acknowledged. First, due to the nature of single-center study, this is a convenience sample and the results of this study do not necessarily represent the prevalence of illicit drug use in all patients with HIV-1 infection in Japan. However, as mentioned above, our clinic treats approximately 15% of the total HIV patients in Japan, and furthermore, most HIV-1 infected patients reside in urban areas such as Tokyo metropolitan area (http://api-net.jfap.or.jp/status/2011/11nenpo/hyo_02.pdf in Japanese). Thus, the discrepancy in the prevalence of illicit drug use between the study patients and all HIV patients in Japan should not be too large. Second, the structured interview method to collect data cannot avoid underreporting of illicit drug usage. Thus, the prevalence of illicit drug use in this population is very likely to be higher than what is reported here. However, underreporting to a certain degree is unavoidable with regard to issues such as illicit drug use [3].

In conclusion, the prevalence of illicit drug use in patients with HIV-1 infection in this large HIV clinic in Tokyo was high at 35%, and was higher in HIV-1 infected MSM (40%). Despite the low prevalence of IDUs (0.5%) among HIV-infected patients reported by the AIDS Surveillance Committee, 5% of patients in this study were IDUs. All relevant parties to the issue of illicit drug use in patients with HIV-1 infection need to recognize that illicit drug use is a huge burden in care and well-being of this population even in Japan, a country with very low prevalence of illicit drug use in the general population. Appropriate measures for prevention and intervention of illicit drug use are urgently needed to ensure proper treatment and prevention of spread of HIV infection.
